# A nanomolecular approach to decrease adhesion of biofouling-producing bacteria to graphene-coated material

**DOI:** 10.1186/s12951-015-0137-x

**Published:** 2015-11-16

**Authors:** Carolina Parra, Fernando Dorta, Edra Jimenez, Ricardo Henríquez, Cristian Ramírez, Rodrigo Rojas, Patricio Villalobos

**Affiliations:** Departamento de Física, Universidad Técnica Federico Santa María, Avenida España 1680, Valparaíso, Chile; Centro de Biotecnología Daniel Alkalay Lowitt, Universidad Técnica Federico Santa María, Avenida España 1680, Valparaíso, Chile; Departamento de Ingeniería Química y Ambiental, Universidad Técnica Federico Santa María, Avenida España 1680, Valparaíso, Chile; Laboratorio de Patología Acuática, Departamento de Acuicultura, Facultad de Ciencias del Mar, Universidad Católica del Norte, Larrondo 1281, Coquimbo, Chile

**Keywords:** Graphene, *Halomonas*, Biofilms, Bacterial adhesion, Antifouling

## Abstract

**Background:**

Biofouling, the colonization of artificial and natural surfaces by unwanted microorganisms, has an important economic impact on a wide range of industries. Low cost antifouling strategies are typically based on biocides which exhibit a negative environmental impact, affecting surrounding organisms related and not related to biofouling. Considering that the critical processes resulting in biofouling occur in the nanoscale/microscale dimensions, in this work we present a bionanotechnological approach to reduce adhesion of biofilm-producing bacteria *Halomonas spp.* CAM2 by introducing single layer graphene coatings. The use of this nanomaterial has been poorly explored for antifouling application.

**Results:**

Our study revealed that graphene coatings modify material surface energy and electrostatic interaction between material and bacteria. Such nanoscale surface modification determine an important reduction over resulting bacterial adhesion and reduces the expression levels of genes related to adhesion when bacteria are in contact with graphene-coated material.

**Conclusions:**

Our results demonstrate that graphene coatings reduce considerably adhesion and expression levels of adhesion genes of biofilm-producing bacteria *Halomonas spp.* CAM2. Hydrophobic-hydrophilic interaction and repulsive electrostatic force dominate the interactions between *Halomonas spp.* CAM2 and material surface in saline media, impacting the final adhesion process. In addition no bactericide effect of graphene coatings was observed. The effect over biofilm formation is localized right at coated surface, in contrast to other antifouling techniques currently used, such as biocides.

## Background

Marine fouling is the accumulation of micro and macroorganisms on underwater surfaces, which provide a favorable mechanism to survive in the environment. The economic impact of fouling on shipping vessels, oceanographic sensors, power plants and aquaculture systems, among others, has been estimated to be in the range of 50 billion euros per year [1–3]. In particular, shipping, fishing and aquaculture industries exhibit extreme fouling cases. On vessels, fouling adds weight to boats and increases hull roughness and hydrodynamic drag, raising fuel consumption by almost 40 %, with the corresponding increase in emissions of greenhouse gases and other pollutants [4]. In aquaculture, settlement of fouling organisms in culture cages causes suffocation of the cultivated species, delaying the time when the cultivated species reaches commercial size [5]. Another singular example is found in boilers cooled with ocean water where the fouling phenomenon causes strong inefficiency in operational parameters and increase in fuel consumption [6].

Current techniques to prevent (antifouling) and fight fouling (fouling-release) include physical (e.g. heat treatments, pulse-power technology, radioactive coatings, flushing, scrubbing and biological control) and chemical methods (e.g. injectable biocides, chlorine, marine bioactive compounds and other form of bactericide coatings such as copper and copper alloys). Although the choice of the right strategy will depend on the cost and application possibilities, antifouling coatings are probably the most cost-effective method for boats and other surfaces. They are typically based on the controlled release of organic solvents into the environment to kill the colonizing microorganism. However this approach offers a non-localized solution, affecting surrounding organisms related and not related to fouling. As a consequence, the use of certain biocides has been restricted by some countries and in Europe a large amount of data has been gathered as part of the Biocidal Products Directive (BPD, 98/8/EC) [7].

The critical processes at the biointerface resulting in biofouling occur in the nanoscale/microscale dimensions: it follows therefore that surface properties which could control biofouling need to be on the same length scales [8]. Search for non-biocidal technology to control the economic and environmental problem caused by biofouling has focused, as a result, on modifying physico-chemical and mechanical properties of surfaces (such as surface free energy, wettability, elasticity and surface topography) at the nanoscale to reduce bacterial attachment [9–14]. As far as coatings for marine antifouling applications are concerned, surfaces with low surface energy or with an optimized surface topography (with patterns in the order of micrometer) have shown promising results [13, 15] opening a new avenue for the development of antifouling coatings.

Graphene is a one-atom thick carbon sheet that has emerged as a new carbon compound with multiple applications in a wide range of industrial processes and products. While graphene is a promising candidate in electronic applications, its use for biological applications, such as antifouling, has been poorly explored. There are many reports of bacterial interaction with graphene oxide (GO), formed by micro- or nano-sized flakes of functionalized graphene in powder, solution or coating [16–18], which induces inactivation of bacterial cells upon direct contact by physical and oxidative damage to cell as its antibacterial mechanism [13, 19–22]. Flake size turns out to be a relevant aspect for the reported antibacterial activity of GO, whether this be in suspension or coating [18, 20]. However in the case of single layer graphene sheets grown on Cu, which as GO are one-atom thick, they have surface areas in the centimeter square range. Hence the mechanism of the bacterial interaction must be different in both cases.

In this report we present a physico-chemical and biological approach to reduce fouling formation in its initial growth stage as biofilm, by introducing graphene coatings that reduce bacterial adhesion to coated surfaces. Nanoscale behavior is discussed in the particular case of biofilm-producing *Halomonas spp.* CAM2, which is used as a model marine bacterium.

## Results and discussion

### Preparation and characterization of nanostructured modified samples

Micro and nanoscale characterization of as-grown graphene on Cu and graphene transferred onto SiO_2_ samples was carried out to evaluate their composition, microstructure, topography with atomic resolution, graphitic quality and contamination.

**Fig. 1:**
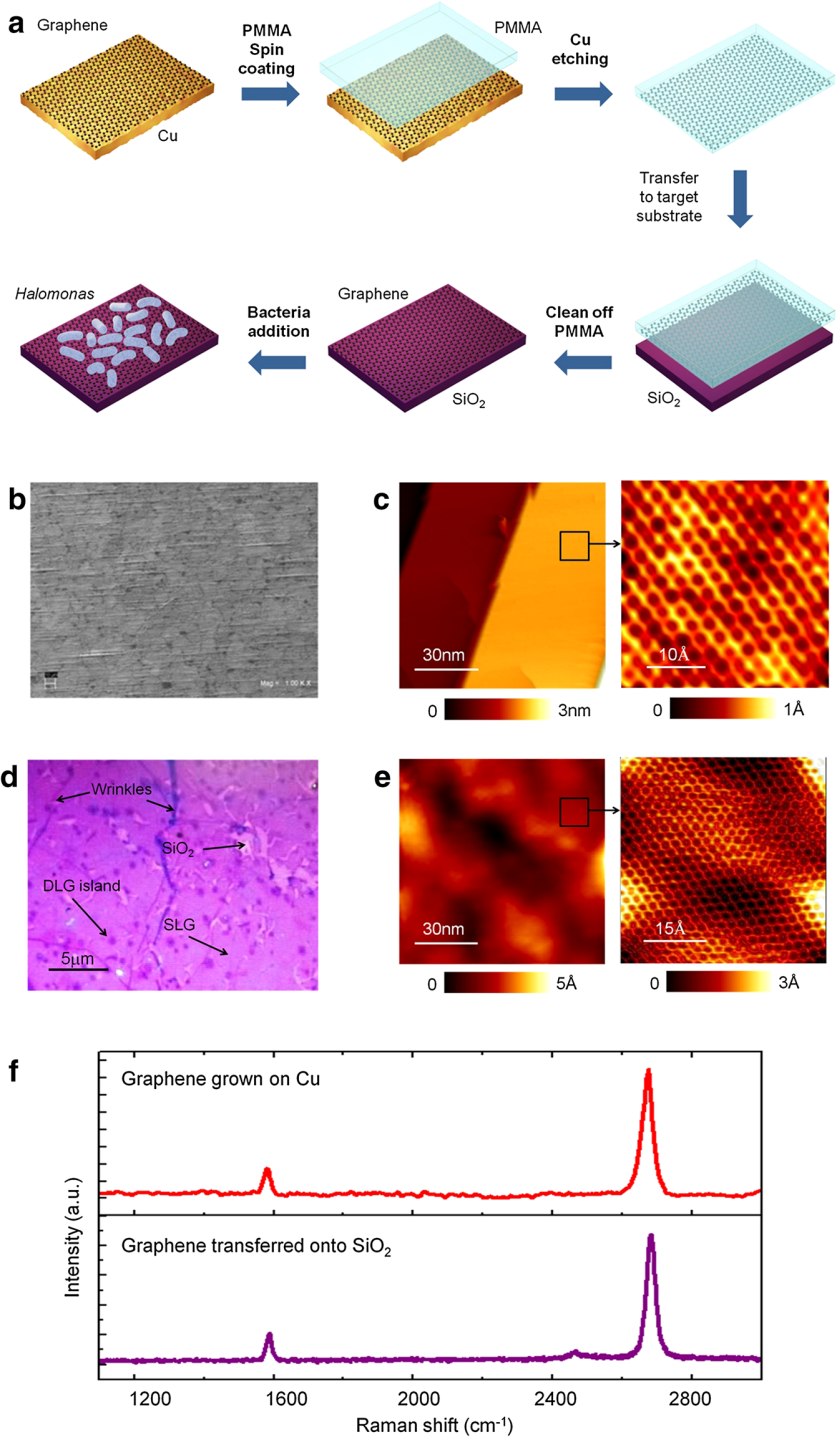
Preparation and characterization of graphene-coated samples. **a** Illustrative diagram showing PMMA-assisted transfer method used to obtain graphene-coated SiO_2_ substrates for present study, **b** SEM image of single layer graphene (SLG) grown on Cu sample, **c **large-scale STM topographic image (100 × 100 nm^2^) of SLG grown on Cu. The filtered atomically resolved image (3.5 × 3.5 nm^2^) shows the hexagonal lattice of SLG, **d** optical microscopy image of SLG transferred onto SiO_2_, **e** STM image of SLG transferred onto SiO_2_ displays honeycomb lattice and **f** representative Raman spectra Raman spectra of SLG grown on Cu (*up*) and SLG transferred onto SiO_2_ (*down*). Background caused by the luminescence of the copper was subtracted in the case of SLG grown and transferred onto Cu. Tunnel current and bias voltages for STM images were between 0.1 and 0.6 nA and 0.1–1 V respectively

Scanning electron micrographs of single layer graphene (SLG) grown on Cu showed some contrast at micrometer scale that could be identified as graphene domains (Fig. 1b). Atomic-resolved images of SLG grown on Cu were obtained by scanning tunneling microscopy (STM) in ultra-high vacuum conditions (Fig. 1c, e). STM topographies exhibit the distinctive honeycomb structure with an interatomic distance of 1.4 A, consistent with literature values [23, 24]. Transfer of graphene to SiO_2_ process is described in materials and methods section.

The morphological characterization of graphene grown on Cu and resulting graphene-coated SiO_2_ samples prior to bacteria contact was screened by SEM, optical microscopy and STM. Wrinkles in CVD graphene grown on Cu are formed by differential thermal expansion, as the metal contracts more than the graphene during post-growth cooling [25]. Such wrinkles are still present after transfer to SiO_2_ substrate, as can be clearly seen in optical microscopy (Fig. 1d).

In addition, different contrast in certain areas is observed which can be identified as bilayer islands on top of a monolayer background. The surface of SLG transferred onto SiO_2_ substrates showed micrometric damages in the graphitic membrane due to the transfer procedure, which leaves some SiO_2_ areas exposed. Clear visualization of the intrinsic hexagonal structure (honeycomb) of graphene transferred onto SiO_2_ was possible by STM (Fig. 1c, e). Few signs of surface contamination were found by this atomic-resolved technique.

To verify the graphitic quality of graphene coatings we performed microRaman spectroscopy measurements. Multiple areas of each sample were analyzed and representative spectra are shown in Fig. 1f. SLG grown on Cu and SLG transferred onto SiO_2_ typically display sharp G (1584 cm-1) and 2D (2680–2693 cm-1) bands, with a small G/2D ratio (0.25 and 0.29 respectively). These results are consistent with single layer graphene, according to values reported in literature [26–28].

### Graphene coating effects on bacterial adhesion

**Fig. 2:**
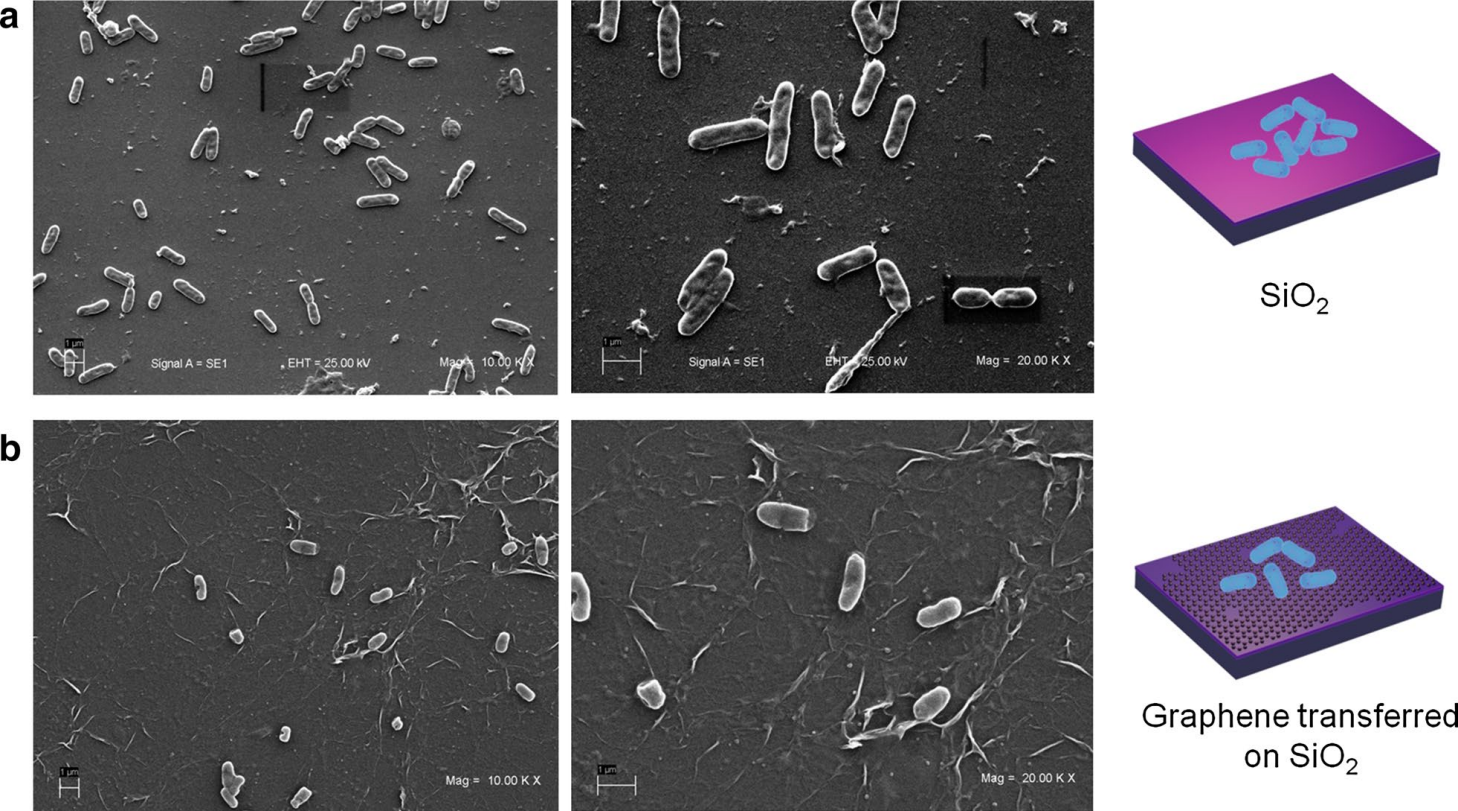
Bacterial attachment to graphene-coated samples. **a** SEM images of *Halomonas spp.* CAM2 after 72 h incubation on SiO_2_ sample and **b** on graphene-coated SiO_2_ samples

We have used SEM and fluorescence microscopy in order to characterize bacterial adhesion to graphene-coated and uncoated SiO_2_. Morphology of *Halomonasspp.* CAM2 incubated for 72 h on SiO_2_ and graphene-coated SiO_2_ samples are shown in Fig. 2a, b, respectively. Intact and smooth cell surfaces were observed for both substrates, in agreement with previous results, confirming the absence of bactericide effects of graphitic coatings [29]. In addition, SEM micrographs show a notorious difference in the bacterial attachment to both surfaces, which is reduced in the case of graphene-coated material (Fig. 2b).

**Fig. 3:**
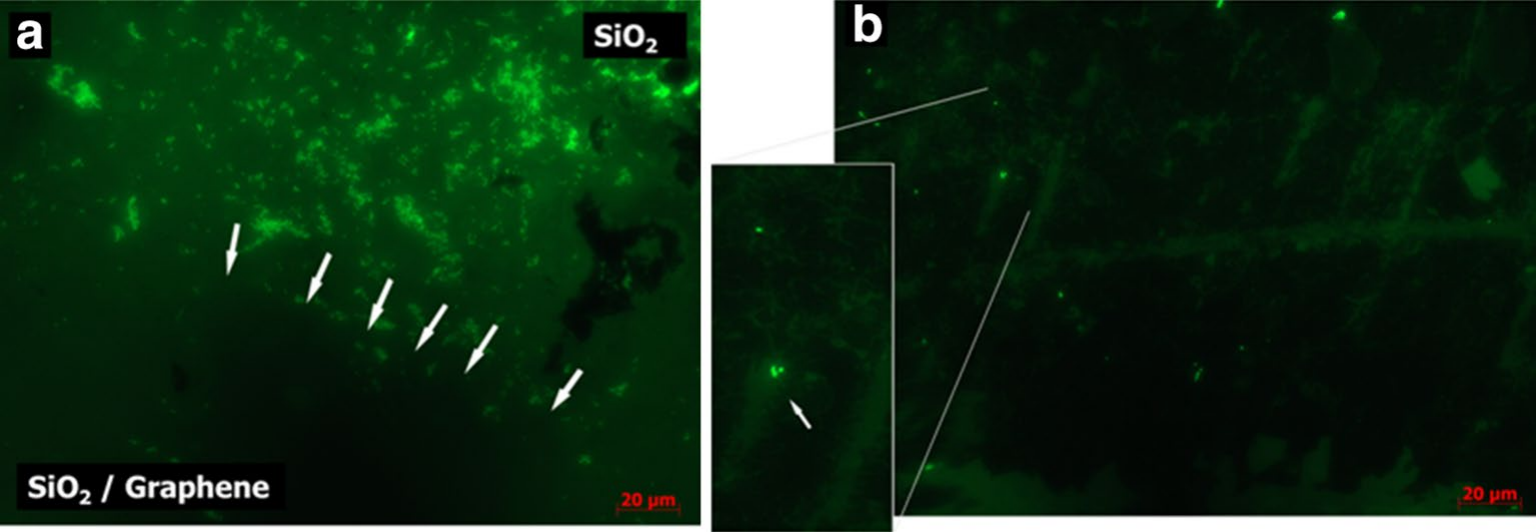
Bacterial distribution in graphene-coated surfaces epifluorescence microscopy image of *Halomonas spp.* CAM2 incubated on partially graphene-coated SiO_2_ surfaces. **a** Partially graphene-coated SiO_2_ surface and **b** completely graphene-coated SiO_2_ surface. *White arrows* in (**a**) are indicating boundary between uncoated and graphene-coated areas on SiO_2_ surface

Representative epifluorescence microscopy images of a partially and completely graphene-coated SiO_2_ samples are shown in Fig. 3a, b respectively. The SiO_2_ substrate was partially coated in order to visualize bacterial attachment in response to coated and uncoated surfaces over the same sample. Bacterial bodies were green stained as indicative of live bacteria on sample surface. Interestingly, the highest concentration of live bacteria was found across the uncoated SiO_2_ surface (upper area in Fig. 3a). In contrast, only few live bacteria were observed across the graphene-coated SiO_2_ area (lower area). For graphene-coated samples (Fig. 3b) the presence of few cells (bright spots in magnified area) can be attributed to the intrinsic micrometer damage of the graphene membrane caused by transfer process (Fig. 1d), which leads to few SiO_2_ exposed areas with increased bacterial attachment. Epifluorescene and SEM results suggest graphene coatings suppress dramatically bacterial attachment which is determinant to biofilm and fouling formation. Such behavior is not related to bactericidal activity, according to same results.

### Graphene coating effects on adhesin gene expression

**Fig. 4:**
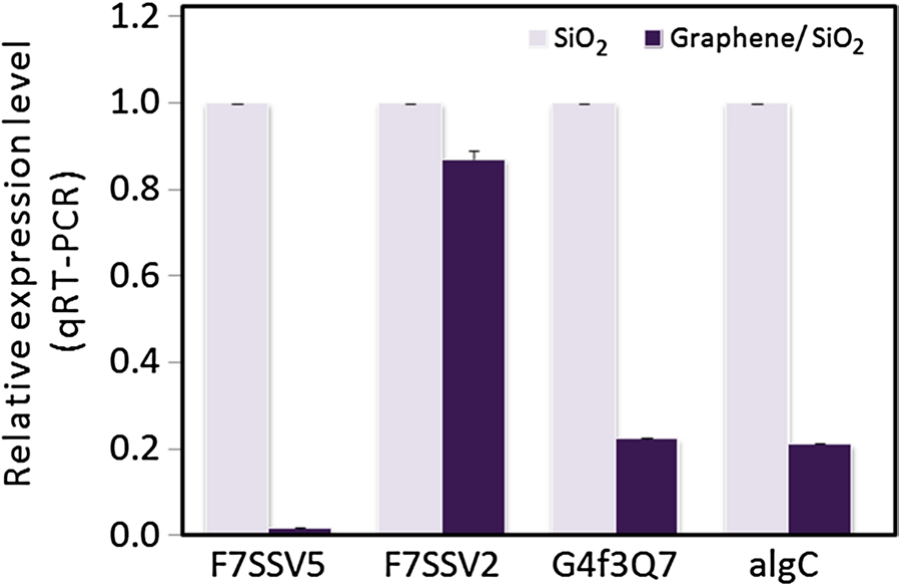
Relative expression levels of 4 adhesin genes. q-PCR results for F7SSV5, F7SSV5, G4f3Q7 and AlgC adhesin genes of *Halomonas spp.* CAM2 incubated on SiO_2_ and graphene-coated SiO_2_ samples

Relative expression of adhesin gene in *Halomonas* CAM2 incubated on SiO_2_ and graphene-coated SiO_2_ samples was evaluated. Quantitative PCR in real time was performed to quantify the expression levels of four selected genes in *Halomonas* CAM2 (Fig. 4). Such genes, F7SSV5, F7SSV5, G4f3Q7 and AlgC, are reported to be related to adhesion in other bacterial species (See methods section for detailed information). F7SSV2 and F7SSV5 genes have been reported to codify adhesin transmembrane proteins in *Halomonas* sp. TD01 [30]. G4f3Q7 codify a polysaccharide intercellular adhesin (PIA) that participates in the biofilm formation in *Halomonas* sp. HAL1 [31]. AlgC has been previously shown to encode phosphomannomutase, which activity produces a precursor for alginate polymerization and biosynthesis of lipopolysaccaride (LPS), both required for biofilm production in *Pseudomonas aeruginosa* [32].

**Fig. 5:**
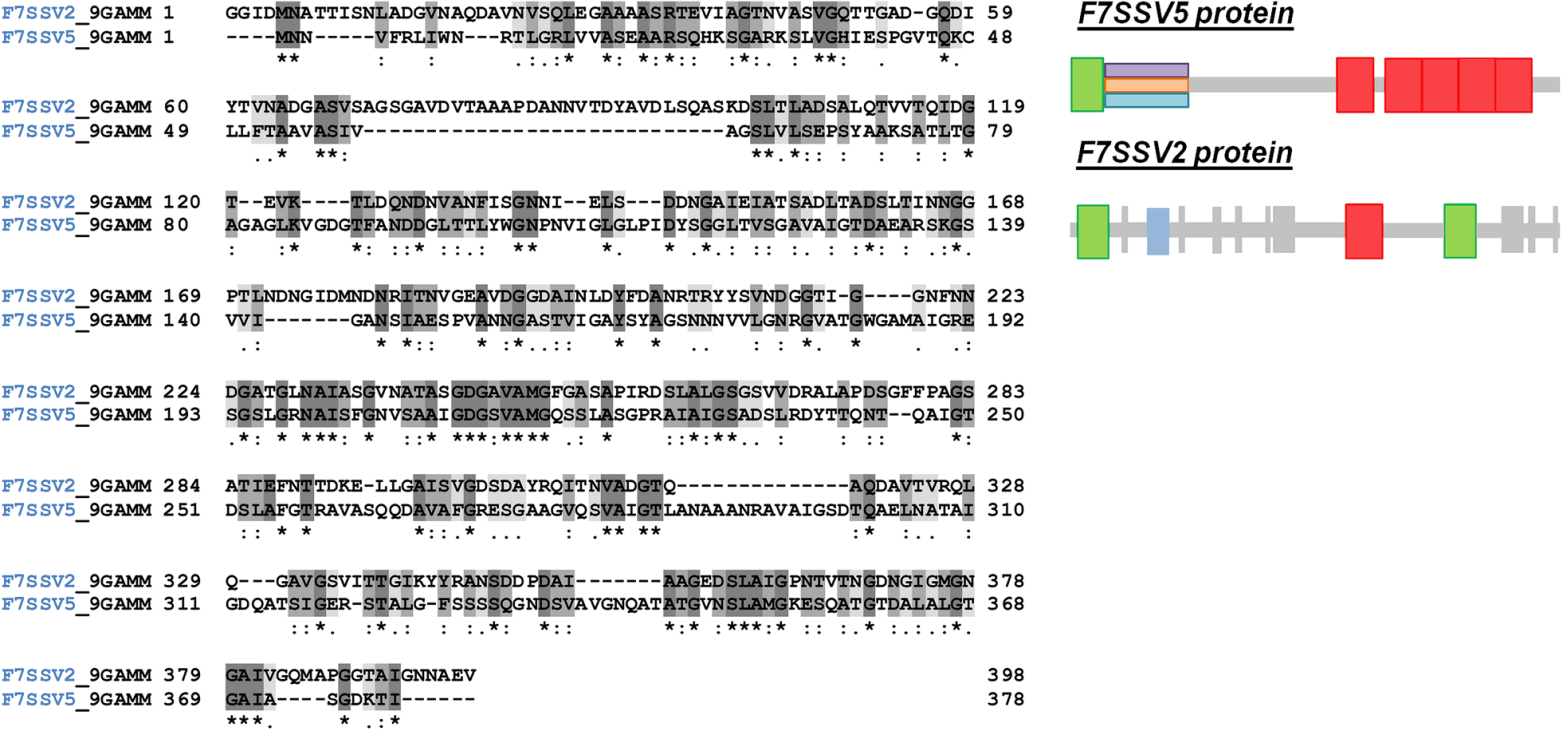
Blast analysis of adhesin genes F7SSV2 and F7SSV5 in *Halomonas*. Arrangement of the Pfam domains in F7SSV5 and F7SSV2 protein. Domains described are based on searching the Pfam-A family against UniProtKB using hmm search. Letters highlighted in gray are indicating amino acid with similarity properties. (Software used UniprotKB Align, http://www.expasy.com)

PCR results in Fig. 4 show the expression levels of F7SSV5, G4f3Q7 and AlgC in *Halomonas spp.* CAM2. They were significantly lower when bacteria were incubated on graphene-coated SiO_2_ samples compared to the corresponding control (uncoated SiO_2_ sample). A mild expression reduction was observed for gene F7SSV2. Such expression difference can be understood in terms of protein location at the cell membrane. Although F7SSV2 and F7SSV5 belong to the same protein family “pfam-A adhesion” and containing similar conserved domain (YadA head; ESPR for Extended Signal Peptide Region), they have different functions, according location of amino acids in their sequence, they have different functions, depending on location of amino acids in their sequence (Fig. 5). F7SSV2 is an integral part of inner cell membrane and is not participating in bacterial adhesion behavior at the same molecular level that F7SSV5 does, which is strongly affected by being an outer membrane protein gene.

### Graphene coating effects on surface energy, wettability and electrostatic interaction

**Fig. 6:**
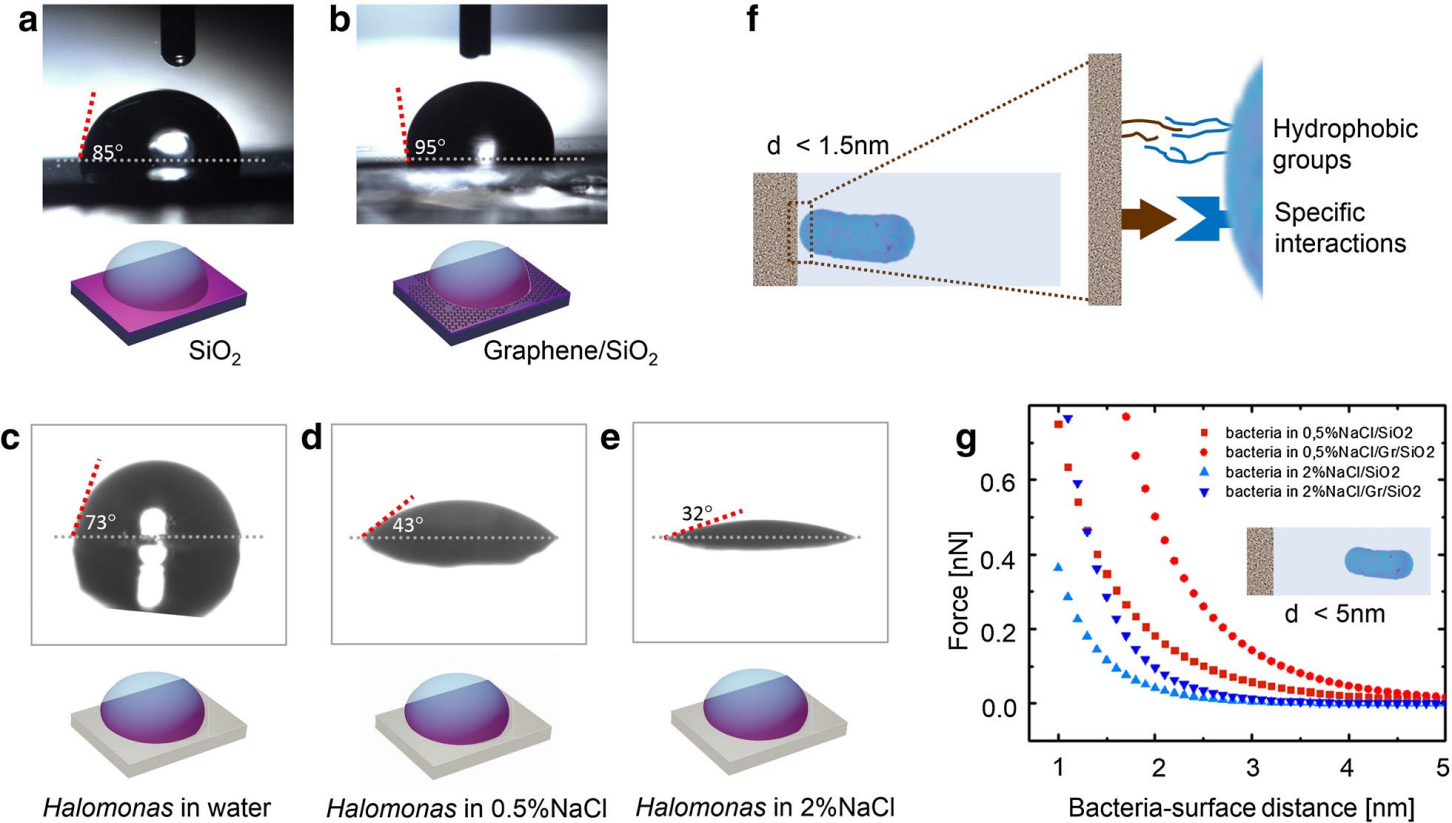
Hydrophobic-hydrophilic nature and electrostatic force between graphene-coated samples and bacteria. Images of contact angle measurements using milliQ water in contact with SiO_2_ (**a**), graphene-coated SiO_2_ (**b**), lawn of *Halomonas spp.* CAM2 previously suspended in water (0 % salinity) (**c**), lawn of *Halomonas spp.* CAM2 previously suspended in 0.5 % NaCl saline solution (**d**) and lawn of *Halomonas spp.* CAM2 previously suspended in 2 % NaCl saline solution (**e**). Diagram (**f**) shows a schematic representation of short-distance interaction (<1.5 nm), originated by physico-chemical forces (like the ones related to surface energy other specific interactions) that are relevant for bacterial adhesion. **g** Theoretical calculations of electrostatic force as function of distance between bacteria and surface (SiO_2_ and graphene-coated SiO_2_) confirm repulsive interaction that increases when material is coated with graphene

Upon approach to a surface, microorganisms will be attracted or repelled, depending on the different non-specific interaction forces [33]. The first relevant interaction in this system is the one related to long-range electrostatic forces between graphene-coated SiO_2_ surface and cells that might be affecting the initial (and reversible) bacterial adhesion process. In graphene-coated SiO_2_, SiO_2_ substrates have a significant surface state density just below the conduction band edge that donates electrons to graphene to balance the chemical potential at the interface. This leads to n-type (or electron-doped) graphene coating [34]. In addition, it has been suggested that bacteria, when introduced into aqueous suspensions, are always negatively charged [35]. To determine if electrostatic long-range interactions between graphene-coated SiO_2_ and bacteria contribute to an initial repulsion between bacteria and graphene-coated substrate we performed theoretical calculations to obtain electrostatic force F(r) between bacteria and material surface (SiO_2_ and graphene-coated SiO_2_) as a function of their separation distance using the expression [36]:

1 $$F\left( r \right) = \frac{{2\pi d_{1} d_{2} \varepsilon \varepsilon_{0} \kappa }}{{d_{1} + d_{2} }}\left( {\frac{{k_{B} T}}{ze}} \right)^{2} \frac{{\phi_{1}^{2} + \phi_{2}^{2} + (2e^{r\kappa } \phi_{1} \phi_{2} )}}{{\left( {e^{r\kappa } + 1} \right)\left( {e^{r\kappa } - 1} \right)}}$$

where *F* is electrostatic force (in N); *r* is distance between bacteria and surface (in m); $d$ is the radius of bacteria (or SiO_2_ piece or graphene-coated SiO_2_) (in m), *ɛ* is the dielectric constant of water [37] (78.43 at 298 K); *ɛ*_0_ is the permittivity of free space (8.854 × 10^−12^C/Jm); *k*_*B*_ is Boltzmann’s constant (1.381 × 10^−23^ J/K), $T$ is temperature (293 K), *z* is the valence of electrolyte ions (1 for NaCl) and *e* is the charge of an electron (1.602 × 10^19^ C). The inverse Debye length *κ* describes the thickness of the electrostatic double layer of counter-ions that surrounds charged parts of the system (bacteria or SiO_2_) in solution. For monovalent electrolytes (e.g. NaCl), *κ*^−1^ is given by 0.304/(*c*)^1/2^ (in 1/nm) where *c* is the concentration of the electrolyte (in mol/L) and contains information of ionic strength of solution [38]. In our case we evaluate 2 and 0.5 % NaCl concentration of suspension media. Surface potential *ϕ* is described by *zeψ*/*k*_*B*_*T*, where *ψ* is the surface potential of the bacteria, SiO_2_ piece or graphene-coated SiO_2_ piece (in V). We considered surface potentials values of SiO_2_ 10 $\mu m$ piece, graphene-coated SiO_2_ 10 *μm* piece and *Pseudomonas* are −35 mV [39] −77 mV [40] and −9 mV [41] respectively. The theoretical force-distance relationship is shown in Fig. 6g. According to this result the electrostatic force in this system is expected to be repulsive and short range (<5 nm for 0.5 % NaCl and <3 nm for 2 % NaCl). Electrostatic repulsion between bacteria and SiO_2_ increases when SiO_2_ is coated with graphene, for both NaCl concentrations, although the effect is higher for lower solution ionic strength.

After overcoming (if) this initial electrostatic repulsion, an even shorter-range hydrophobic interaction (generally when bacteria-surface distance is smaller than 1.5 nm [28]) have a strong impact on bacterial surface adhesion, a characteristic that is mainly determined by physico-chemical surface properties [42].

In order to determine the influence of possible hydrophobic characteristics of graphene coatings over bacterial adhesion, we performed contact angle measurements on SiO_2_ (Fig. 6a), graphene-coated SiO_2_ (Fig. 6b) and lawns of *Halomonas spp.* CAM2 previously suspended in water (0 % NaCl) and saline buffer (0.5 % NaCl and 2 % NaCl) (Fig. 6c, d, e respectively).

Contact angles are related to the surface free energies [43]. Hydrophobic coatings are often used to minimize adhesion since they create a larger contact angle between the bacteria’s glue and the surface. This results in less wettability (lower surface energy) and less fouling since the adhesive is not being able to spread across the surface [44]. According to our measurements a transition from hydrophilic surface (contact angle of ~85° ± 0.7) for SiO_2_ substrate to hydrophobic surface (contact angle of ~95° ± 0.3) for graphene-coated SiO_2_ is observed.

In addition, cells that possess a hydrophilic character attach preferentially to hydrophilic surfaces (large surface energy), whereas hydrophobic cells prefer hydrophobic surfaces [3, 45]. The hydrophobic-hydrophilic nature of *Halomonas spp.* CAM2 surface changes as the bacterium grows in different media. Reported response of *Halomonaselongata* to saline NaCl media, like the one used in the current experiments, displays an enhanced hydrophilicity [46], which makes the cell more attractive to water molecules in the environment and prevents desiccation. Our contact angle results confirmed such hydrophilic nature of *Halomonas spp.* CAM2 and a clear trend to increased cell hydrophilicity as a function of salinity of suspension media; 73° for 0 %, 43° for 0.5 % and 32° for 2 % NaCl (Fig. 6c, d, e respectively). In addition these results suggest that the modification from hydrophilic to hydrophobic nature of graphene-coated SiO_2_ determines the suppression of bacterial attachment for hydrophilic *Halomonas spp.* CAM2. These results show that both interactions, electrostatic (<5 nm range) and hydrophobic-hydrophilic (<1.5 nm range), are presumably affecting the bacterial attachment process, causing a notorious decrease in adhesin gene expression of *Halomonas spp.* CAM2, with the corresponding reduction of bacterial adhesion to graphene-coated surfaces.

## Conclusion

In this paper we present a nano-biotechnological approach to decrease the attachment of marine bacteria *Halomonas spp.* CAM2 by introducing graphene coatings. According to our theoretical and experimental results graphene coatings modify surface energy and electrostatic interactions with bacterial cells which determines an important reduction of bacterial adhesion, a relevant parameter involved in biofilm formation and consequent biofouling emergence. This nanoscale surface modification affects the expression of genes related to adhesion that are notoriously decreased when bacteria are in contact with graphene-coated SiO_2_ surfaces instead of uncoated SiO_2_ surfaces.

No bactericide effects of graphene-coated SiO_2_ were observed. Such behavior indicates the effect over biofilm formation is localized right at coated surface, in contrast to other antifouling techniques currently used, such as biocides, that exhibit negative effects over all surrounding aquatic species, not necessarily related to biofilm or biofouling formation. We expect this work will contribute to provide new opportunities for designing effective and environmentally friendly antifouling surfaces based on nanoscale modified materials.

## Methods

### Materials

Silicon dioxide coated wafers and commercial CVD graphene grown on Cu was obtained from Graphene Supermarket Company. Graphene and SiO_2_ samples used in this study were 1 cm^2^ in area.

### Preparation and characterization of Gr/SiO2

Transfer of graphene films grown on Cu to SiO_2_ substrates was achieved by the poly(methylmethacrylate) (PMMA) assisted method as shown in Fig. 1a. Thin layer of PMMA on graphene on Cu foil was produced by spin-coating. The polymer provides a supportive framework for graphene before the transfer. The underneath Cu substrate is then etched away by an ammonium persulfate ((NH_4_)_2_SO_8_) solution. After the Cu foil is completely dissolved, the floating membrane can be scooped and placed on SiO_2_. After drying, the polymeric film is dissolved with acetone.

PMMA and ammonium persulfate ((NH_4_)_2_SO_8_) used for graphene transfer procedure were purchased from Sigma Aldrich.

Scanning Tunneling Microscopy (UHV-VT Omicron) was used to characterize nanoscale morphology. Sample preparation before STM measurements consists of 200 °C annealing in UHV for 30 min. Platinum-iridium tips were used for all STM measurements. The experimental data were analyzed by using WSxM software. Scanning Electron Microscopy (SEM) images were recorded using a Carl Zeiss microscope (EVO MA-10) to characterize microscale morphology and qualitative bacterial adhesion. MicroRaman measurements (Renishaw, 532 nm laser) were used to characterize quality of as-grown graphene and transferred graphene onto SiO_2_. Contact angle measurements were performed to characterize surface hydrophobicity of coated and uncoated Cu samples. A drop of milliQ water (2μL) was placed on the surface of graphene-coated SiO_2_ and uncoated samples and images were immediately captured using a high-resolution camera. Bacterial hydrophobicity was measured following standard methods [41] with some modifications. A bacterial strain suspended in 40 mL of water, 0.5 % NaCl and 2 % NaCl were filtered on a micropore cellulose nitrate filter (pore size 0.45 μm, Sartorius Stedim Biotech, Germany) by filtration of the suspension using negative pressure. The filters with a bacteria film were dried at room temperature during 90 min in order to obtain a stable water contact angle measured by sessile drop method using 1 μL of distilled. The contact angle was measured based on image analysis [47] using the image processing software Image J with the plug-in Drop Shape Analysis based on B-spline snakes algorithm developed by [48].

### Bacterial strain isolation

A marine strain *Halomonas spp.* CAM2 was used in the biofilm formation assays. Bacteria was previously isolated from illness larvae of the Chilean scallop *Argopecten purpuratus* Lamarck, 1891 (bivalvia, pectinidae) and characterized [49]. Stock cultures of the CAM2 strain were maintained at 4 °C on Tryptic soy agar (Difco) supplemented with NaCl (2 %), and subcultured every 2 weeks. For long-term preservation, CAM2 strain cultures were frozen at −80 °C in Tryptic soy broth (Difco) supplemented with 2 % NaCl (w/v) and 20 % glycerol (v/v) [50]. When required, frozen cultures were recovered by streaking onto Tryptic soy agar plates (Difco) supplemented with NaCl (2 %), which were incubated at 20 °C for 24 h.

### Bacteria culture and exposure to Gr/SiO_2_

Surfaces used for biofilms growth were sterilized by rinsing several times with ethanol and sterilized DI water. Bacterial adhesion assays on partially graphene-coated SiO_2_ were performed in petri dishes. TSA Agar plates were inoculated with freshly growing cells of *Halomonas spp.* CAM2, so that a lawn of bacteria was grown. After 48 h incubation at 25 °C a half of the surface containing bacteria (in exponential to early stationary phase) was harvested and suspended in 10 ml of sterile saline buffer (0.5 % NaCl). This volume was poured on pieces of 1 cm^2^ of Gr/SiO_2_ and SiO_2_ and incubated for 72 h at 20 °C. An aliquot was removed in order to determine the cell concentration by dilution plating. All experiments were run in triplicate.

### Bacterial adhesion study

Analysis of bacterial adhesion was conducted in order to evaluate morphology and viability of microorganisms. For SEM characterization bacteria were fixed on samples with 3 % (v/v) glutaraldehyde and dehydrated by washing with a graded ethanol series (from 10 to 100 %), followed by critical-point drying and gold coating.

Distribution of bacteria on Gr/SiO_2_ or SiO_2_ surfaces was determined directly in situ. For epifluorescence analysis Gr/SiO_2_ or SiO_2_ pieces were submerged into solution provided by standard LIVE/DEAD BacLight bacterial viability kit (0.01 mM of Syto9 and 0.06 mM of propidium iodide). Samples were kept dark during 15 min and then observed by epifluorescence microscopy Olympus × 71.

### Expression of adhesion genes

*Halomonas spp.* CAM2 has not been previously sequenced. Because this strain was only recently isolated, no genes participating in adhesion for this bacterial specie have been identified and sequenced. Genes F7SSV5, F7SSV2 and G43U7, which have been described to participate in adhesion of *Halomonas elongata* [30] together with AlgC, involved in the same process for *Pseudomonas aureginosa,* were used as target. Primers were designed using sequences from GenBank and ApliX software V3.1. Sequence for each gene is described in the Table 1.

**Table 1:** Sequences of oligonucleotide primers used for qPCR

Gene name	sequence	Amplicon size (bp)
16S-F	5-TCGCGTTAACTTCGCCACAA-3	184
16S-R	5-AGCGGTGAAATGCGTAGAGA-3	
F7SSV5-F	5-ATGTCGCCTGATCACCGATA-3	197
F7SSV5-R	5-TGACTCGCTCGCTTTTGGTA-3	
F7SSV2-F	5-TTTGCTGGCTTGGCTGAGAT-3	187
F7SSV2-R	5-AGTCATTGCTGGCACAAACG-3	
G4F3Q7-F	5-ACCAGATCGGCAAGCACAAA-3	176
G4F3Q7-R	5-TGGCTGGCGTTTTCATCCAA-3	
algC-F	5-TGATCTTCGACGTCAAGTGC-3	153
algC-R	5-AAATGTGACCGCTCATCTCG-3	

Primers were designed for this study using AmplifX V1.3.7 software with genes published on genebank

For RNA isolation, SiO_2_ surfaces with and without graphene were washed twice with phosphate buffer. Bacteria were scrapped from samples surface with a sterile cotton swab and were stored at −80 °C awaiting RNA isolation [51]. Bacterial RNA was extracted using TRIzol reagent (Ambion by Lifetechnologies).

Samples were defrosted, and genes were extracted using chloroform. RNA was recovered by precipitation with ethanol 70 % and was load on Qiagen RNeasy minElute spin column following manufacturer’s instructions.

RNA quantity was determined by measuring the absorbance at 260 nm using a Nanodrop spectrophotometer ND-1000 (Thermo Fisher Scientific). Contaminated genomic DNA was removed by TURBO DNA-free kit (ambion by lifetechnologies). Further, total RNA samples were analyzed for the presence of DNA contamination by qRT-PCR using 16S rRNA target.

Purified RNA was converted to cDNA using RevertAid™ first strand cDNA synthesis kit (Fermentas) with random hexamer primers according to manufacturer’s instructions.

Real time PCR was performed according to the protocol of the SYBR Green/Rox qPCR Master Mix (Fermentas) with Stratagene Mx3000P real-time PCR system (Stratagene); 16S mRNA levels were used for normalization.

Amplification courses in all genes included the following 3 steps: (1) 1 cycle of an initial denaturation for 10 min at 95 °C, (2) 40 cycles of an initial denaturation for 30 s at 95 °C. Annealing for 1 min at 55 °C and extension for 30 s at 72 °C and, (3) 1 cycle of an initial denaturation for 1 min at 95 °C, annealing for 30 s at 55 °C a final extension for 30 s at 95 °C.

Relative quantification of each target gene (F7SSV5, F7SSV2, G4F3Q7, AlgC) encoding for adhesin in each experimental sample, and control versus reference gene (rDNA16S) was performed in according with Livak Eq. 1: relative expression ratio (R) = 2^−[ΔCt sample − ΔCt control]^ = 2^−ΔΔCt.^
